# Global research trends of immunotherapy and biotherapy for inflammatory bowel disease: a bibliometric analysis from 2002 to 2021

**DOI:** 10.1186/s12938-022-01011-9

**Published:** 2022-06-27

**Authors:** Jia-Qi Xiong, Yun-Feng Fu, Jian-Hao Qiu, Wang-Di Liao, Ling-Yu Luo, Si-Hai Chen

**Affiliations:** 1 Department of Gastroenterology, The First Affiliated Hospital of Nanchang University, Yongwaizhengjie Street 17th, Donghu District, Nanchang, Jiangxi China; 2 Nanchang University, Nanchang, Jiangxi China

**Keywords:** Bibliometric analysis, Biological therapy, Immunotherapy, Inflammatory bowel disease

## Abstract

**Background:**

It is known that inflammatory bowel disease is the result of a defective immune system, and immunotherapy and biological therapy have gradually become important means to treat it. This paper focused on the bibliometric statistical analysis of the current research progress to summarize the research status of this field and analyze the research trends in recent years.

**Methods:**

Two visualization tools, CiteSpace and VOSviewer, were used to explore the data of journals, institutions, countries/regions, authors, references, and keywords for the literature included in the Web of Science Core Collection from January 1, 2002, to December 31, 2021.

**Results:**

A total of 312 papers were published in 120 journals by 603 institutions from 40 countries/regions, with 9463 co-cited references. The United States has the most publications with the highest total citations in the world. Inflammatory Bowel Diseases published the maximum number of papers, and Gastroenterology devoted the most co-citations to immunotherapy and biological therapy for IBD. In addition, we found that the studies before 2009 mostly focused on clinical trials while researchers have paid more attention to clinical management in therapy for IBD since 2009. Combination therapy and management of the treatment for the disease have become research hotspots.

**Conclusion:**

The focus of immunotherapy and biotherapy for IBD has shifted from clinical trials to the management of the risks and benefits of immunotherapy.

**Supplementary Information:**

The online version contains supplementary material available at 10.1186/s12938-022-01011-9.

## Introduction

Inflammatory bowel disease (IBD) includes Crohn’s disease (CD) and ulcerative colitis (UC), which are characterized by chronic inflammation of the gastrointestinal tract. 5-Aminosalicylic acid (5-ASA) may be chosen as initial therapy for those with mild ulcerative colitis who value the safety of 5-ASA more than its low efficacy, but 5-ASA should not be used to induce and maintain remission in moderate-to-severe or recalcitrant ulcerative proctitis and should be upgraded to biologic or immunomodulatory agents for treatment [1, 2]. Although the etiology and pathogenesis of IBD are not fully understood, inflammatory responses caused by abnormal intestinal mucosal immune system responses are known to play an important role in the pathogenesis of IBD [3]. The peak incidence of CD and UC occurs between the ages of 20 and 30 years while the second peak of ulcerative colitis occurs between 60 and 79 years of age [4]. IBD as a chronic and difficult disease to cure can significantly affect the individuals’ quality of life and cause a high financial burden [5]. Immunotherapy and biological therapy for inflammatory bowel disease have gradually become the research direction among peer experts. In particular, the emergence of biologic therapies has significantly improved clinical and endoscopic outcomes, as well as hospitalization rates and surgical-related morbidity in IBD [6]. The publications also have increased during the past two decades.

Bibliometrics is the use of statistical methods to analyze books, articles, and other publications, especially scientific content. It is often used to track the relationship between citations in academic journals [7]. CiteSpace and VOSviewer are bibliometric visualization tools that are widely used to visualize and analyze emerging trends and transition patterns in scientific literature. Although there was a review in 2014 that summarized the temporal trend of the number and type of yearly published articles from 1993 to 2013 [8], it did not further analyze the relationship among authors, countries/regions, publishing institutions, references, keywords, etc. An article published in 2020 using text-mining techniques that studied the trend of IBD gives us an understanding of the current state of IBD research [9]. To gain insights into research trends and hotspots in the field of immunotherapy and biotherapy for IBD, we performed a bibliometric analysis of articles from the Web of Science Core Collection (WoSCC) which provides comprehensive information and data covering the vast majority of papers published in the field of immunotherapy and biologic treatment of inflammatory bowel disease by using CiteSpace and VOSviewer.

## Results

### Annual publications

**Fig. 1:**
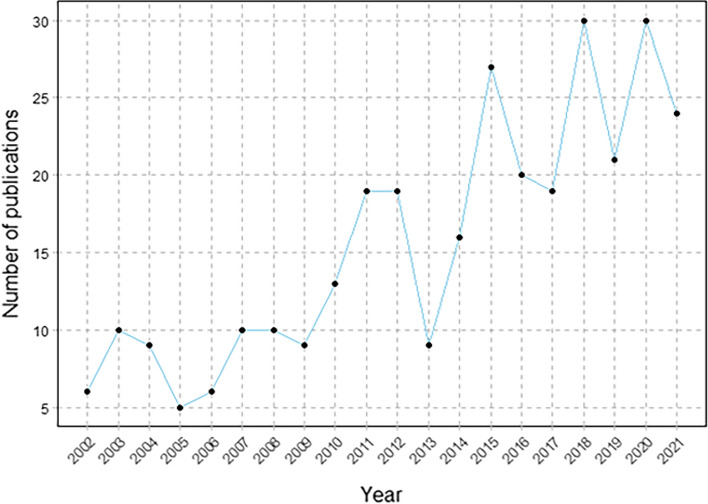
Number of publications per year

In total, 312 records were included, and the number of publications by year is presented in Fig. 1. Most studies were articles (199/312, 63.78%), followed by reviews (66/312, 21.15%). Other article types include 7 editorial materials, 16 letters, and 2 proceedings papers. In Fig. 1, we find that the number of published papers showed an upward trend on the whole during the past 20 years; although the numbers slightly declined in some years (2005, 2013, 2017, 2019), they recovered in the following years.

### Journals and co-cited academic journals

A total of 312 publications related to immunotherapy and biological therapy for IBD were published in 120 academic journals. Five journals published more than 10 articles, and the journal with the most publications was Inflammatory Bowel Diseases (*n* = 48, IF2021 = 5.325), followed by the Journal of Crohn’s & Colitis (*n* = 13, IF2021 = 9.071) and American Journal of Gastroenterology (*n* = 11, IF2021 = 10.864). Three of them are located in the United States, except for the Journal of Crohn’s & Colitis, which was in the Netherlands. The top 21 productive journals with citations, countries, and impact factors are listed in Table 1.

**Table 1:** The top 21 most productive journals

Journals	Documents	Citations	IF2021	Country
Inflammatory Bowel Diseases	48	1319	5.325	United States
Journal of Crohn’s & Colitis	13	471	9.071	Netherlands
American Journal of Gastroenterology	11	948	10.864	United States
Clinical Gastroenterology and Hepatology	11	687	11.382	United States
Digestive Diseases	11	121	2.404	Switzerland
World Journal of Gastroenterology	8	79	5.742	Peoples R China
Alimentary Pharmacology & Therapeutics	7	367	8.171	England
Gastroenterology	7	554	22.628	United States
International Journal of Colorectal Disease	7	104	2.571	Germany
Scandinavian Journal of Gastroenterology	7	170	2.423	Norway
Digestive and Liver Disease	6	130	4.088	Italy
Zeitschrift Fur Gastroenterologie	6	26	2.000	Germany
Digestive Diseases and Sciences	5	114	3.199	United States
Gut	5	691	23.509	England
Journal of Clinical Gastroenterology	5	140	3.062	United States
Bmc Gastroenterology	4	44	3.067	England
Current Opinion In Gastroenterology	4	73	3.287	United States
European Journal of Gastroenterology & Hepatology	4	81	2.566	United States
Journal of Gastroenterology And Hepatology	4	94	4.029	Australia
Journal of Pediatric Gastroenterology and Nutrition	4	88	2.839	United States
Revista Espanola de Enfermedades Digestivas	4	16	2.086	Spain

IF2021: impact factor in 2021

When two or more journals are simultaneously cited by one or more later publications, it is said that these journals constitute a co-citation relationship. Among 1605 co-cited academic journals, Gastroenterology (*n* = 1562, IF2021 = 22.628) ranked first weighted by co-cited journals, followed by Inflammatory Bowel Diseases (*n* = 871, IF2021 = 5.325) American Journal of Gastroenterology (*n* = 852, IF2021 = 10.864), and Gut (*n* = 796, IF2021 = 23.509). Among the co-cited journals, The New England Journal of Medicine has the highest Impact Factor (IF2021 = 91.245).

### Authors and co-cited authors

**Fig. 2:**
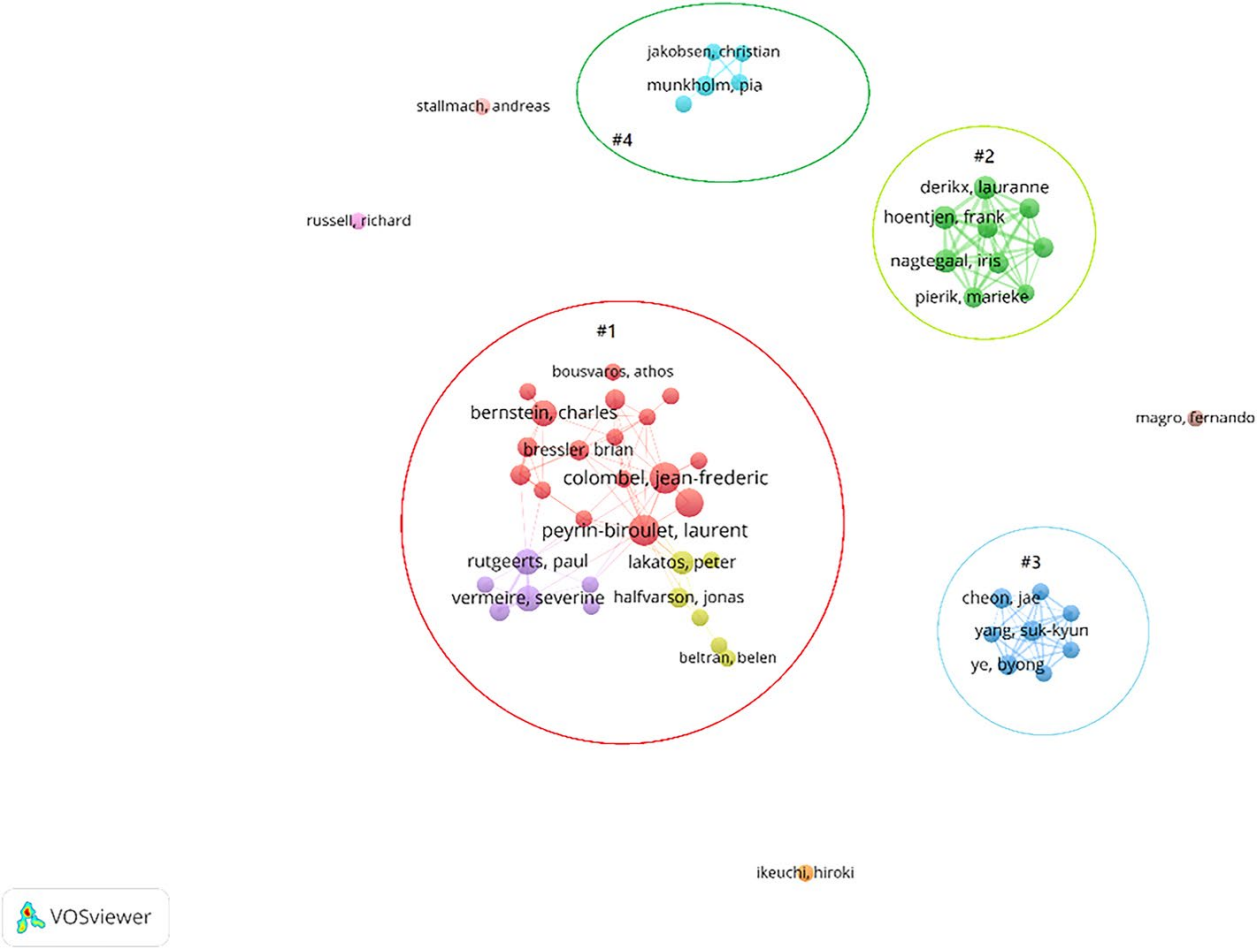
The cluster map of the top 55 productive authors

A total of 1656 authors were involved in immunotherapy and biological therapy for IBD studies from 312 publications. Three authors published at least seven articles. Colombel, Jean-Frederic, and Peyrin-Biroulet, Laurent tied for first place with the most publications (*n* = 8), followed by Beaugerie, Laurent (*n* = 7). The authors (55/1656, 3.32%) with a publication number greater than or equal to three (*T* = 3) were used to construct the network map of the top 55 cooperatively productive authors (Fig. 2). Among these authors, four major groups of related projects contribute to most of the world’s publications. The node sizes of Colombel, Jean-Frederic, Peyrin-Laurent, and Beaugerie, Laurent are larger owing to their greater numbers of publications. They are all in the same group (#1), indicating significant cooperation among them.

When two authors’ papers are simultaneously cited by a third author’s papers, it is said that the two authors are co-cited. If the co-citation frequency of these two authors is higher, it indicates that they work on similar topics and that their academic relationship is closer. Among 6451 co-cited authors, four authors had co-citations over 110. Sandborn, William had the most co-citations (*n* = 251) and ranked the first, followed by Colombel, Jean-Frederic (*n* = 143), Rutgeerts, Paul (*n* = 116).

### Countries/regions and organizations

**Fig. 3:**
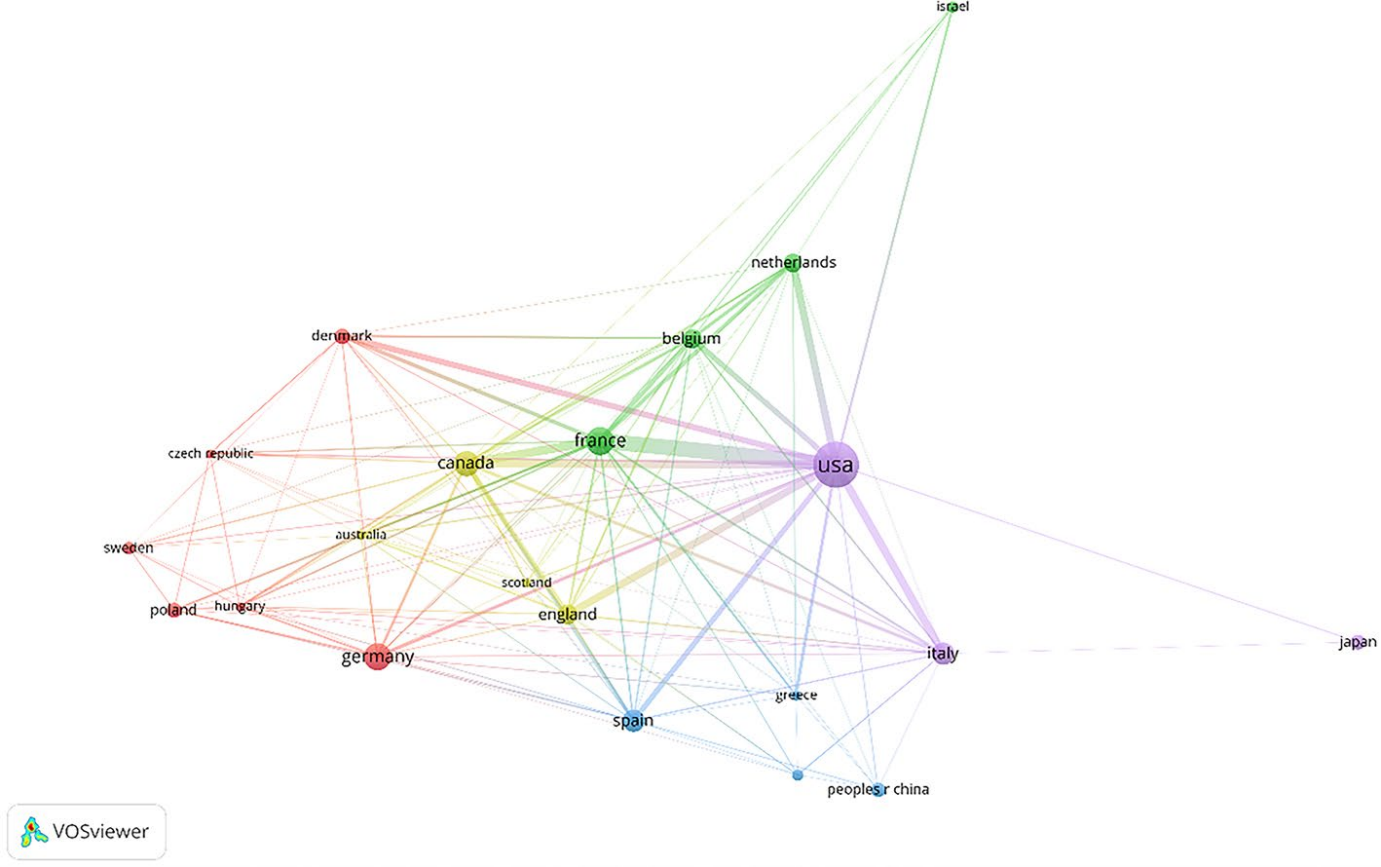
Co-citation map of the top 21 productive countries/regions

A total of 312 publications were coauthored by 603 organizations from 40 countries/regions. The top 10 countries/regions are distributed on two continents (North America and Europe), eight of which are distributed in Europe. The top-ranked country is the USA (*n* = 75), with more than twice as many as the second, France (*n* = 33), followed by Germany (*n* = 32) and Canada (*n* = 28). In the top 20 list, there are three countries or regions in Asia, including Japan (*n* = 12), China (*n* = 11), and South Korea (*n* = 8). The countries/regions (21/40, 56.96%) with a publication number greater than or equal to five (*T* = 5) were used to construct the network (Fig. 3). In this network map, the USA, Germany, and France have larger node sizes, representing more publications. Different countries/regions have diverse networks. For example, the United States has close cooperation with France, Canada, Italy, Spain, Italy, and the Netherlands; France has cooperation with the USA, Canada, Belgium, the Netherlands, England, etc.

**Fig. 4:**
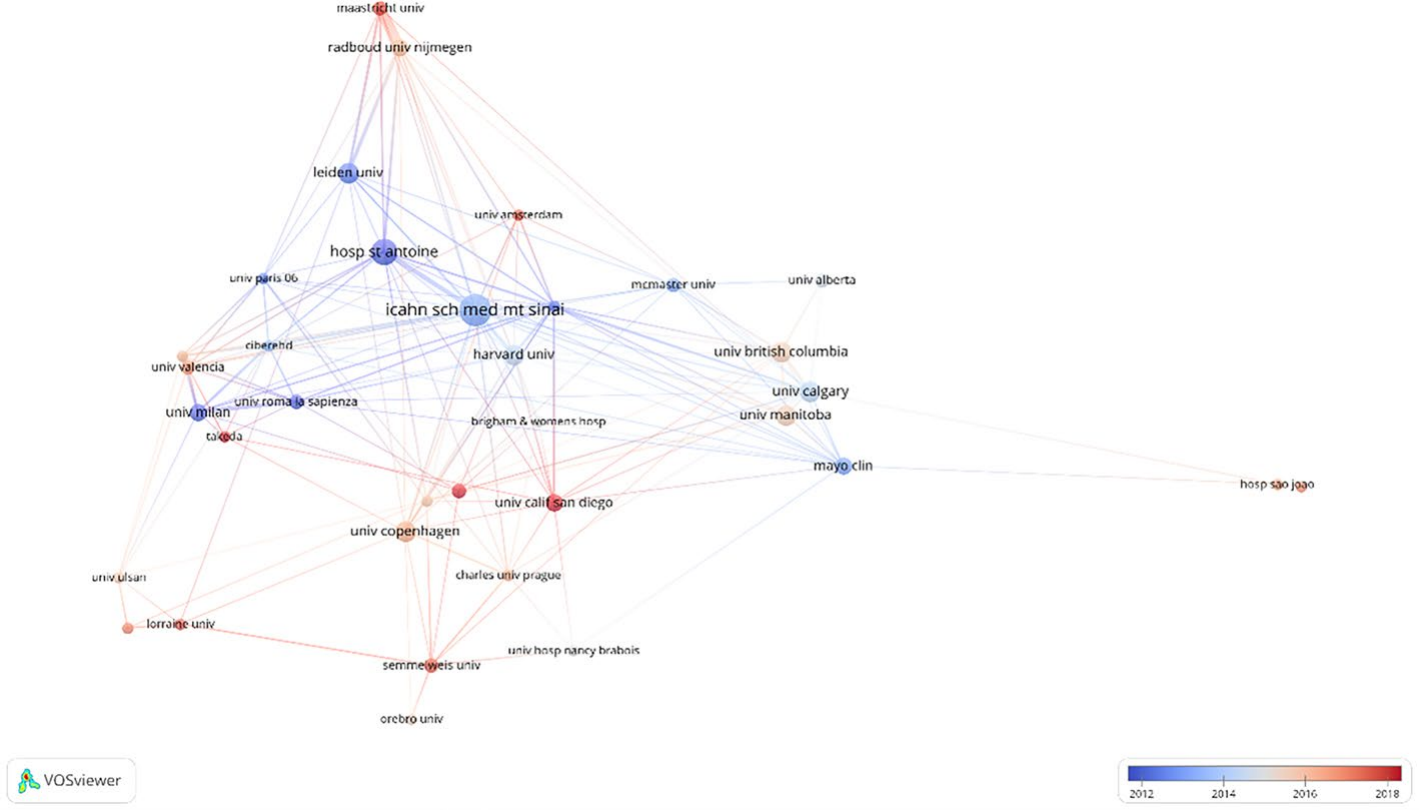
The overlay visualization of the largest set of organizations

Icahn School of Medicine at Mount Sinai (*n* = 11, USA) takes first place according to the number of publications, followed by Hôpital Saint-Antoine (*n* = 9, France) and Harvard University (*n* = 7, USA). The top 10 institutions are distributed in five countries on two continents, more than half of which are located in North America. Detailed information is listed in Table 2. In terms of citations, Harvard University ranks first with 615 citations, followed by Icahn School of Medicine at Mount Sinai, which has 547 citations, and Mayo Clinic, which has 544 citations, all of which are located in the United States. The top 10 organizations with the most citations are listed in Table 3. Organizations (32/603, 5.3%) with more than or equal to four (*T* = 4) publications were used to construct the network map and overlay visualization, and the largest subnetwork was presented (Fig. 4). Within this visual network, the nodes of the Icahn School of Medicine at Mount Sinai were the largest because of its publications. Many active collaborations were noted among different institutions. For example, the Icahn School of Medicine at Mount Sinai has close cooperation with Harvard University, Hôpital Saint-Antoine, the University of Calgary, and the University of Manitoba. Furthermore, the overlay visualization (Fig. 4) also showed that four institutions have been active in immunotherapy and biological therapy for IBD in recent years, including Maastricht University, University of California, San Diego, and Cleveland Clinic.

**Table 2:** Top 10 organizations with the most documents

Organization	Documents	Citations	Country
Icahn School of Medicine at Mount Sinai	11	547	USA
Hôpital Saint-Antoine	9	461	France
Harvard University	7	615	USA
University of Calgary	7	298	Canada
University of Copenhagen	7	203	Denmark
University of British Columbia	7	172	Canada
University of Manitoba	7	94	Canada
Mayo Clinic	6	544	USA
University of California, San Diego	6	310	USA
University of Milan	6	133	Italy

**Table 3:** Top 10 organizations with the most citations

Organization	Documents	Citations	Country
Harvard University	5	615	USA
Icahn School of Medicine at Mount Sinai	11	547	USA
Mayo Clinic	6	544	USA
Dartmouth Hitchcock med ctr	4	515	USA
University Hospital Leuven Gasthuisberg	3	492	Belgium
Hôpital Saint-Antoine	9	461	France
HeiligHartziekenhuis	1	433	Netherlands
Imeldaziekenhuis	1	433	Belgium
Massachusetts General Hospital	3	414	USA
Lille University Hospital Center	2	405	France

### Co-cited references and reference citation bursts

When two or more papers are cited by one or more later papers at the same time, the two papers are said to constitute a co-citation relationship, which can be called co-cited references. The greater the number of co-cited references, the greater the correlation between references. There was a total of 9463 cited references among 312 publications. The top 10 co-cited references are listed in Table 4. All of these journals are from four top journals: Lancet, The New England Journal of Medicine, Gastroenterology, and the Journal of Crohn’s and Colitis. Hanauer, Stephen et al. published an article titled “Maintenance infliximab for Crohn’s disease: the ACCENT I randomised trial” in Lancet, which ranked first in the list with the most instances of co-citation times (*n* = 47). This study performed a randomized controlled trial to prove the efficacy of maintenance infliximab therapy in those with active Crohn’s disease who respond to a single infusion of infliximab, and this treatment was safe and well tolerated [10]. The second most co-cited reference (*n* = 40) was a prospective observational cohort study written by Beaugerie, Laurent et al. with the title “Lymphoproliferative disorders in patients receiving thiopurines for inflammatory bowel disease: a prospective observational cohort study” in 2009, which confirms the increased risk of developing lymphoproliferative disorders in those treated with thiopurines for IBD [11]. The references (44/9463, 0.46%) with co-citations greater than or equal to 15 (*T* = 15) can be divided into four clusters. Cluster 1 consists of 14 papers, the articles of which focus on the risk and management of inflammatory bowel disease [11–13]. Cluster 2 also contains 14 articles that pay attention to the risk of lymphoma associated with therapy of inflammatory bowel disease [14–16]. Cluster 3 is composed of 9 studies that center on the efficacy of various monoclonal antibodies for Crohn’s disease. Only 7 articles belong to Cluster 4, which focuses their research on combination therapy for inflammatory bowel disease [17–19]. The details of the top 44 co-references are shown in Additional file 1: Table S4.

**Table 4:** The top 10 co-cited references

Title	Citation	Authors^a^	Pub. year	Journals
Maintenance infliximab for Crohn’s disease: the ACCENT I randomised trial	47	Hanauer, Stephen	2002	Lancet
Lymphoproliferative disorders in patients receiving thiopurines for inflammatory bowel disease: a prospective observational cohort study	40	Beaugerie, Laurent	2009	Lancet
Infliximab, Azathioprine, or Combination Therapy for Crohn’s Disease	38	Colombel, Jean-Frederic	2010	The New England Journal of Medicine
Infliximab for Induction and Maintenance Therapy for Ulcerative Colitis	33	Rutgeerts, Paul	2005	The New England Journal of Medicine
European evidence-based Consensus on the prevention, diagnosis and management of opportunistic infections in inflammatory bowel disease	31	Rahier, Jean-Francois	2014	Journal of Crohn’s and Colitis
Adalimumab for Maintenance of Clinical Response and Remission in Patients With Crohn’s Disease: The CHARM Trial	29	Colombel, Jean-Frederic	2007	Gastroenterology
Risk Factors for Opportunistic Infections in Patients With Inflammatory Bowel Disease	29	Toruner, Murat	2008	Gastroenterology
Epstein–Barr virus-positive lymphoma in patients with inflammatory bowel disease treated with azathioprine or 6-mercaptopurine	27	Dayharsh, Gerald	2002	Gastroenterology
Infliximab Maintenance Therapy for Fistulizing Crohn’s Disease	26	Sands, Bruce	2004	The New England Journal of Medicine
A Short-Term Study of Chimeric Monoclonal Antibody cA2 to Tumor Necrosis Factor α	24	Targan, Stephan	1997	The New England Journal of Medicine

^a^The first author

**Fig. 5:**
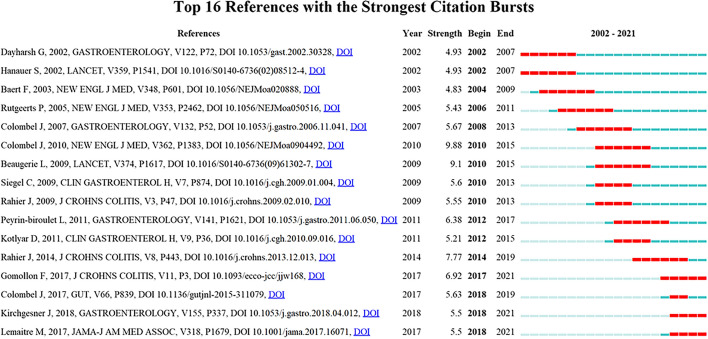
The top 16 references with the strongest citation bursts

CiteSpace provides burst detection to detect large changes in citations during a period, which can be used to discover the decline or rise of research topics. We detected bursts between 2002 and 2021 based on an analysis of the 312 publications. The top 16 publications with the strongest citation bursts are presented in Fig. 5. The year column in the figure indicates the year references published. In addition, the begin and the end columns indicate the start and end time of the outbreak. The blue line represents the timeline from 2002 to 2021, with red overlays representing the periods in which keyword burst occurs. The two ends of the red line indicate the starting year and the ending year, between which the duration of the outbreak is indicated. Two references with citation bursts were published by Dayharsh, Gerald and Hanauer, Stephen in 2002, which attracted much attention as soon as they were delivered, with citation bursts that continued until 2007. In recent years, two references have experienced citation explosions, which are still hot research topics. These two studies research the risk of infections [20] and lymphoma [21] associated with thiopurines and anti-tumor necrosis factor (TNF) agents, used alone or in combination, providing important evidence for the management of IBD.

### Co-occurrence keywords and citation bursts

**Fig. 6:**
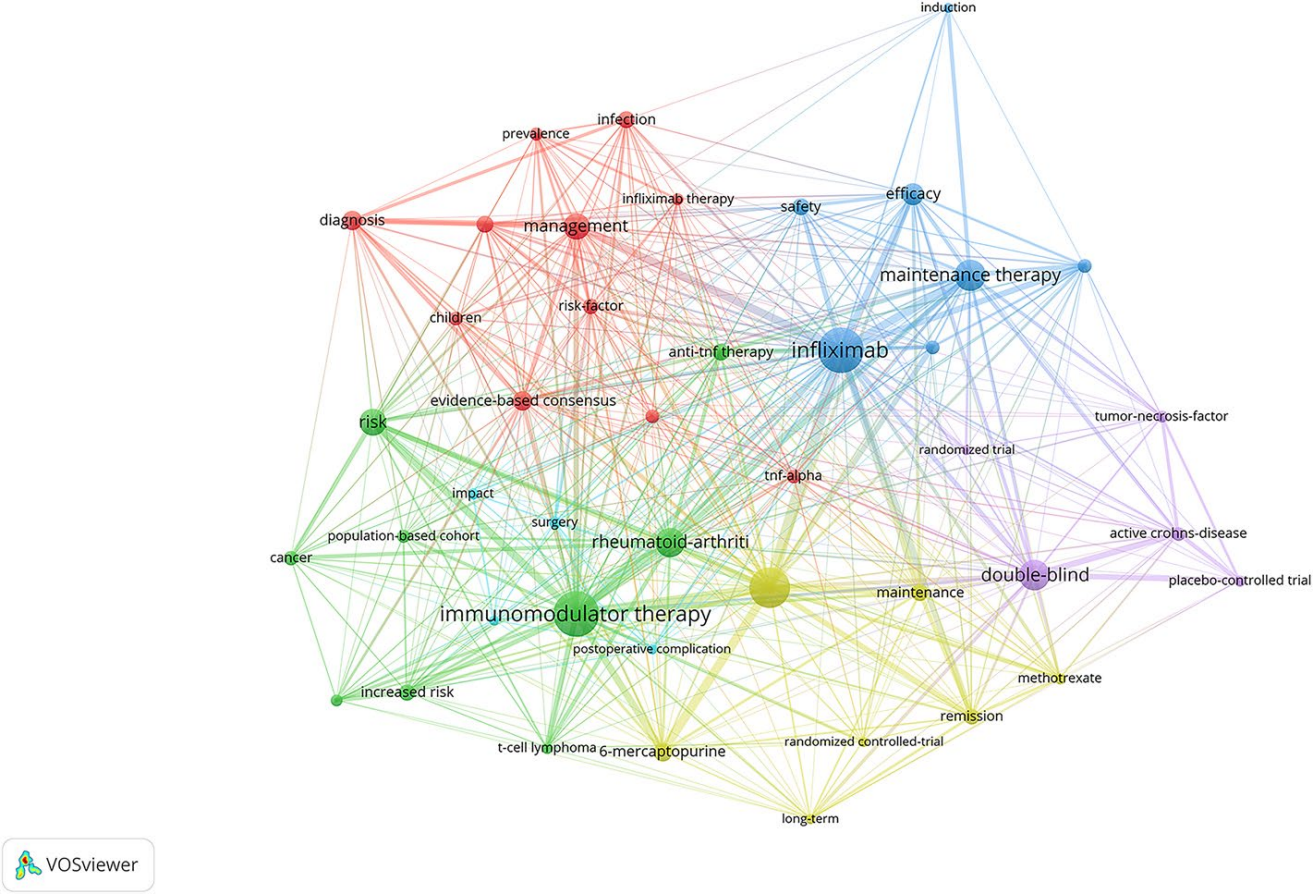
The co-occurrence map of keywords by frequency

A total of 806 keywords were extracted in VOSviewer after merging synonymous keywords in 312 publications for subsequent analysis. After excluding four keywords out of 47 keywords with a keyword co-occurrence number greater than or equal to 10, 43 (43/806, 9.06%) keywords were formed six clusters (Fig. 6). The main keywords for Cluster 1 are “management”, “diagnosis” and “evidence-based consensus”, which represented clinical management and related research. The keyword with the maximum number of co-occurrences in Cluster 2 is “immunomodulator therapy”, and the cluster mainly focuses on methods of immunotherapy and their risks. Cluster 3 is mainly about keywords related to the treatment of infliximab, including “infliximab”, “maintenance therapy”, “efficacy” and “safety”. The most important keyword in Cluster 4 is “azathioprine”, and this cluster mostly studies purine analogs and methotrexate. Cluster 5 is about trial and Cluster 6 pays attention to surgery for inflammatory bowel disease. These keywords are shown in Additional file 1: Table S5.

**Fig. 7:**
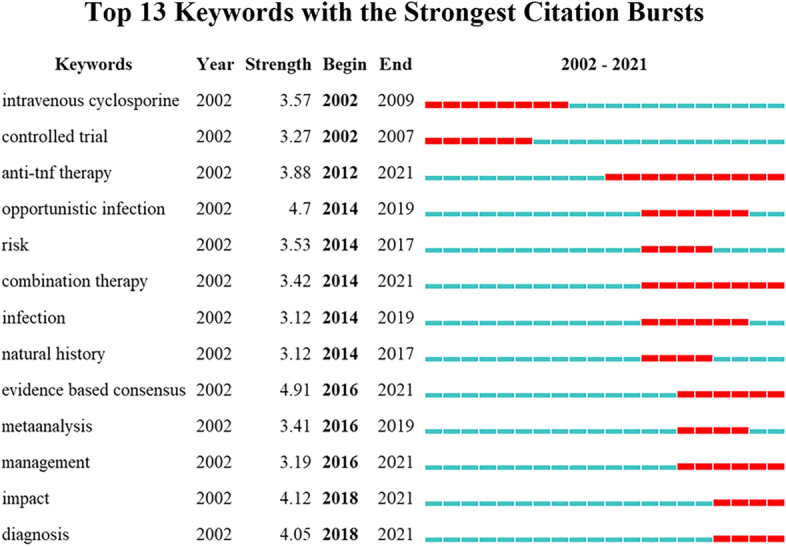
The top 13 keywords with the strongest citation bursts

We used CiteSpace to detect keyword bursts based on an analysis of the 312 papers. The results in Fig. 7 are the strongest citation bursts of the top 13 keywords. The column Year means all keywords have appeared in 2002, which means that these keywords were already present before 2002. However, the outbreak time and duration of the keywords were different. The two keywords “intravenous cyclosporine” and “controlled trial” were already in the outbreak stage in 2002, then “intravenous cyclosporine” ended the outbreak in 2009 while “controlled trial” ended in 2007. We further conducted a quick analysis using the same search method on WoSCC for relevant literature from 1992 to 2011 and found that the outbreak of “controlled trial” started in 2002 while the “controlled trial” started in 2000. “anti-TNF therapy” had a citation outbreak in 2012. Two years later, “combination therapy” and the risk of therapy such as infection began to citation burst. The hotness of the keywords “opportunistic infection” and “infection” subsided until 2019, while “anti-TNF therapy” and “combination therapy” remained in citation bursts until 2021. In addition, four keywords that have been the hot spots of research in recent years are “evidence-based consensus”, “management”, “impact” and “diagnosis”.

## Discussion

In this study, we performed a literature search in the WoSCC and included 312 articles published between 2002 and 2021 after excluding literature not relevant to immunotherapy and biological therapy for IBD. Before 2010, the number of papers published each year was no more than 10, and the total number of papers was only 65, which means the development of this field is slow. After 2010, the annual number of publications increased rapidly, especially between 2013 and 2016. This may be related to the publication of relevant consensus on the management of inflammatory bowel disease [22, 23]. Before 2010, immunotherapy and biological therapy for inflammatory bowel disease was still in clinical trials [18, 24].

A total of 312 articles were published in 120 journals, among which Inflammatory Bowel Diseases and the Journal of Crohn’s & Colitis were dedicated to inflammatory bowel disease and contributed the most articles. Immunotherapy and biotherapy have also been published in many top journals such as The Lancet and The New England Journal of Medicine, indicating that immunotherapy and biotherapy are important means for the treatment of IBD and have attracted the attention of academia.

Among 1656 authors, Colombel, Jean-Frederic, from the Icahn School of Medicine at Mount Sinai, an authoritative institution in this field in the United States, has published the most articles. In the top 10 co-cited papers analyzed subsequently, we found two papers from Colombel, Jean-Frederic, published in Gastroenterology in 2007 [25] and 2010 [17], published in The New England Journal of Medicine, and the impact factor of these two articles totaled more than 110 points, which is enough to indicate their activity and strong influence in the field. The top five organizations with citations are mostly in the United States, so it is not strange that the number of co-citations in the United States is by far the greatest in the world. Two other bibliometrics articles also acknowledge this, even though their subject is inflammatory bowel disease and ours is immunotherapy and biotherapy for inflammatory bowel disease [9, 26]. The top 20 countries in terms of publication volume are all developed countries except China. The top 10 institutions with the number of publications and citations are all located in developed countries. This may be related to the fact that IBD is more prevalent in developed countries [27]. As the incidence of inflammatory bowel disease in developing countries increases and receives increasing attention, there will be much research output in developing countries in the future [4, 28]. This hypothesis could be a direction for future research to confirm the correlation between regional research output and penetrance of IBD in a population.

In the co-cited literature analysis, we can get a glimpse of the research topics in the field of immunotherapy and biotherapy of IBD and their changes over time. Among the relevant studies in this field, the top 16 publications with the strongest citations show a significant change in research trends over time. Five studies published from 2005 to 2007 were all clinical trial articles related to treatment, and the articles that appeared after 2009 mainly focused on two aspects: (1) evidence-based consensus in IBD [22, 23, 29]; (2) prospective cohort studies [11, 30], meta-analyses [31, 32] and systematic reviews [33] about the risk of immunotherapy and biological therapy. This also reflects a research trend after the emergence of a new treatment method, which means that the use of immunological agents and biologics to treat IBD has become more and more clinically mature, and now consensus and management of the disease and balance the risks and benefits of treatment are the main research direction in this field, which is also consistent with the following keyword analysis. The early keywords intravenous cyclosporine, anti-tumor necrosis factor, and controlled trial are all about the treatment of inflammatory bowel disease, while “evidence-based consensus”, “management”, “impact” and “diagnosis” are related to the diagnosis, management, prognosis of the disease.

This is the first study to conduct a bibliometric analysis to systematically analyze immunotherapy and biological therapy for IBD, which can guide clinicians and academics in this field. Compared to traditional narrative reviews, the bibliometric analysis provides better insight into research focuses and trends.

Like other bibliometric research, our study has several limitations. First, all of our original data were retrieved from WoSCC, while records from other databases, such as PubMed or Scopus, have not been studied; thus, the possibility of omitting relevant articles remains. However, Web of Science (WOS) is a professional database of information containing a variety of characteristics that can be used for bibliometric research, which is something that PubMed data cannot do because it cannot do citation analysis and co-citation analysis [34]. WOS and Scopus are two different types of databases and have different calculation methods, but the evaluation indexes of both are highly relevant [35, 36]. To a certain extent, we suppose this work can reflect the general situation and general trend of this field. Second, although we manually conducted filtering in original records to exclude those less relevant to our study, there may still be a selection bias. Third, we cannot rule out the possibility of author homophones and signature variants because author information cannot be accurately obtained through existing tools. Fourth, we used two software packages during the analysis, and even on the same data, some reasonable and unavoidable differences between CiteSpace and VOSviewer may also exist. Finally, we did not include data for 2022 due to insufficient data.

## Conclusion

In this study, we used CiteSpace and VOSviewer to analyze publications on immunotherapy and biological therapy for IBD in the past 20 years. Inflammatory Bowel Diseases, with the most publications, and Gastroenterology, with the most co-citations, were the most significant journals for immunotherapy and biological therapy for IBD. Colombel, Jean-Frederic has made outstanding contributions in this field because he published numerous articles and was co-cited frequently in publications. The United States contributed the most to immunotherapy and biological therapy for IBD, and Harvard University had the most citations. This bibliometric study offers a comprehensive understanding of references and keywords, which discover that the focus of immunotherapy and biological therapy for IBD has shifted from clinical trials to the management of the risks and benefits of immunotherapy.

## Methods

### Data source and search strategy

We searched articles published in the last 20 years using the Web of Science Core Collection on March 6, 2022. The search was performed using the following keywords and terms: (TS=(“Inflammatory Bowel Disease” OR “ulcerative colitis” OR “crohn disease”) AND (KP=(immunotherapy OR “immun* therapy” OR “immun* treat*” OR biotherap* OR “biological treat*”) OR AK=(immunotherapy OR “immun* therapy” OR “immun* treat*” OR biotherap* OR “biological treat*”) OR TI = (immunotherapy OR “immun* therapy” OR “immun* treat*” OR biotherap* OR “biological treat*”) AND (PY==("2021" OR "2019" OR "2018" OR "2020" OR "2017" OR "2016" OR "2015" OR "2014" OR "2013" OR "2012" OR "2011" OR "2010" OR "2009" OR "2008" OR "2007" OR "2006" OR "2004" OR "2003" OR "2005" OR "2002")) NOT (DT==("MEETING ABSTRACT")). Each article was downloaded in the form of “full records and cited references.” The information included the title, keywords, author, institution, country, abstract, and references.

### Inclusion and exclusion criteria

We included the records without language restrictions. The exclusion criteria were as follows: (a) duplicate reports; (b) conference abstracts; (c) IBD mentioned in the abstract but not in the text; (d) only talking about inflammatory bowel disease or immunotherapy; (e) only talking about molecular mechanisms; and (f) mainly talking about other diseases.

### Analysis tools and analysis processes

CiteSpace (version 6.1. R1 Basic, Drexel University, Philadelphia, PA, USA) and VOSviewer (version 1.6.9, Leiden University, Leiden, Netherlands) are bibliometric analysis visualization software for visualizing and analyzing the networks. We used CiteSpace V to detect the references and the keywords with the strongest citation bursts. VOSviewer was used for clustering analysis of authors, the cooperation between countries/regions, and institutions. In addition, we also used it to analyze co-cited journals, co-cited authors and co-cited references, and co-occurrence keywords to explore authoritative journals, important literature, core authors, and research hotspots in this field.

We downloaded 705 articles from WoSCC and finally retained 312 publications that were used for analysis in this study after manual screening according to the above exclusion criteria and then imported the data into CiteSpace and VOSviewer for bibliometric analysis and constructed visualization maps. Because of differences in the algorithms of the two software packages, the keywords extracted by them did not all match, so we merged the keywords found by the two software packages. We merged synonyms and different variants of the same keyword, and the relevant documents are in Additional file 1: Tables S1 and S2. The criteria for merging are as follows: (1) Singular and plural forms of the same word, (2) Different spellings of the same word, (3) Different words expressing the same meaning. We also excluded four keywords, “inflammatory bowel disease”, “therapy”, “crohns-disease” and “ulcerative-coliti”, which we thought were meaningless for exploring research trends in this study. In the process of organizational analysis, we found that some institutions changed their names or had variations in their names, resulting in a different spelling of the name of the same institution. For this reason, we merged some of the institution names, and the merged names are listed in Additional file 1: Table S3.

## Supplementary Information

**Additional file 1: Table S1.** Keywords merged in CiteSpace. **Table S2.** Keywords merged in VOSviewer. **Table S3.** The merged names of organizations in VOSviewer. **Table S4.** Top 44 co-cited publications showed by clusters. **Table S5.** Top 43 co-occurrence keywords showed by clusters.

## Data Availability

All data and materials used to support this study are available through reasonable requests from the corresponding authors.

## Abbreviations

IBD: Inflammatory bowel disease
CD: Crohn’s disease
UC: Ulcerative colitis
5-ASA: 5-Aminosalicylic acid
WoSCC: Web of Science Core Collection
WOS: Web of Science
TNF/tnf: Tumor necrosis factor
