# Supplementary material for: Global research trends of immunotherapy and biotherapy for inflammatory bowel disease: a bibliometric analysis from 2002 to 2021
Source: Biomed Eng Online. 2022 Jun 27;21:42. doi: 10.1186/s12938-022-01011-9 (PMC9238098; doi:10.1186/s12938-022-01011-9)
Supplement: Supplementary file 1 — Additional file 1: Table S1. Keywords merged in CiteSpace. Table S2. Keywords merged in VOSviewer. Table S3. The merged names of organizations in VOSviewer. Table S4. Top 44 co-cited publications showed by clusters. Table S5. Top 43 co-occurrence keywords showed by clusters. [file 12938_2022_1011_MOESM1_ESM.docx]

### Additional file 1

**Table S1. Keywords merged in CiteSpace**

| The keyword used in CiteSpace | The merged keywords |
| --- | --- |
| tumor necrosis factor | tnf |
| antitumor necrosis factor | anti-tumor necrosis factor |
| anti-tumor necrosis factor-alpha | anti tnf alpha |
| anti tnf therapy | anti-tnf therapy |
|  | factor alpha therapy |
|  | necrosis factor therapy |
| azathioprine therapy | azathioprine treatment |
| biological therapy | biological treatment |
| cancer | carcinoma |
|  | malignancy |
| cdp870 | certolizumab pegol cdp870 |
| children | pediatric patient |
| combination therapy | combined therapy |
| crohn disease | crohns disease |
|  | crohns-like disease |
| disease | disorder |
| epstein barr virus | epstein barr |
|  | epstein-barr virus |
| human herpesvirus 8 | hhv 8 |
| hodgkins lymphoma | hodgkins disease |
| hpv infection | human papillomavirus infection |
| immunomodulator therapy | immunomodulatory therapy |
|  | receiving immunomodulator therapy |
| immunosuppressive therapy | immunosuppressive treatment |
| individual medicine | personal medicine |
| infliximab maintenance | maintenance infliximab |
| infliximab therapy | infliximab treatment |
| injury | lesion |
| kappa b | nuclear factor-kappa b |
| lymphoproliferative disease | lymphoproliferative disorder |
| maintenance therapy | maintenance treatment |
| medical therapy | medical treatment |
| non hodgkin lymphoma | non hodgkins lymphoma |
|  | non-hodgkins lymphoma |
| posttransplant lymphoproliferative disease | posttransplantation lymphoproliferative disorder |
| recurrence | relapse |
| resistance | resistant |
| septic shock | shock - septic |
| surgery | surgical treatment |
| tumor necrosis factor-alpha | tnf alpha |
| transplant | transplantation |
| ulcerative coliti | ulcerative colitis (uc) |
| 5 aminosalicylate therapy | 5 aminosalicylate use |
| 5 aminosalicylic acid | 5-aminosalicylic acid |
| adalimumab | monoclonal antibody adalimumab |
| airway hyperresponsiveness | bronchial hyperreactivity |
| cd4 monoclonal antibody | anti cd4 monoclonal antibody |
| inflammatory bowel disease | inflammatory bowel disease (ibd) |
|  | ibd |
|  | ibd patient |
| therapy | treatment |

**Table S2. Keywords merged in VOSviewer**

| The keyword used in VOSview | The merged keywords |
| --- | --- |
| 5-aminosalicylate therapy | 5-aminosalicylate use |
| adult patient | adult |
| adalimumab | monoclonal-antibody adalimumab |
| anti-tnf therapy | tnf therapy |
|  | anti-tnf immunotherapy |
|  | anti-tnf treatment |
|  | necrosis factor therapy |
| antibody therapy | antibiotic-therapy |
| anti-tnf | antitumor necrosis factor |
| azathioprine therapy | azathioprine treatment |
| c virus-infection | c infection |
| cancer risk | cancer-risk |
| cancer | malignancy |
|  | cancers |
| cdp870 | certolizumab pegol cdp870 |
| combination therapy | co-treatment |
| consensus guideline | consensus document |
| controlled trial | controlled-trial |
| crohns-disease | crohn |
| disease | disorder |
| epstein-barr-virus | epstein-barr |
| evidence-based consensus | evidence based consensus |
| hemophagocytic syndrome | hemophagocytic lymphohistiocytosis |
| herpes-virus | herpesvirus |
| hodgkins-lymphoma | hodgkins-disease |
| hpv infection | human-papillomavirus infection |
| immune checkpoint inhibitor | immune checkpoint blockade |
| immunosuppression | immune suppression |
| immunomodulator therapy | receiving immunomodulator therapy |
|  | immunomodulatory therapy |
|  | immunosuppressive therapy |
|  | immunotherapy |
| inflammatory bowel disease | ibd |
|  | ibd patient |
|  | inflammatory bowel-disease |
|  | inflammatory-bowel-disease |
| infliximab therapy | infliximab treatment |
| lymphoproliferative disease | lymphoproliferative disorder |
| maintenance therapy | maintenance treatment |
| tnf-alpha | necrosis-factor-alpha |
| non-hodgkin-lymphoma | non-hodgkins-lymphoma |
| nonmelanoma skin cancer | nonmelanoma skin-cancer |
| children | pediatric-patient |
| pneumocystis-carinii pneumonia | pneumocystis-carinii-pneumonia |
| posttransplant lymphoproliferative disease | posttransplantation lymphoproliferative disorder |
| prevention | prophylaxi |
| propria t-lymphocyte | propria t-cell |
| randomized trial | randomized-trial |
| resistance | resistant |
| surgical treatment | surgical-treatment |
| tuberculosis | tb |
| t-cell | t-lymphocyte |
| thiopurine therapy | thiopurine treatment |
| toll-like receptor | to-target |
| transplant | transplantation |
| tumor-necrosis-factor | tnf |

**Table S3. The merged names of organizations in VOSviewer**

| The names of organizations used in VOSview | The merged names |
| --- | --- |
| icahn sch med mt sinai | mt sinai sch med |
| univ naples federico ii | univ naples federico 2 |
|  | univ naples 2 |
| jeroen bosch hosp | jeroen bosch ziekenhuis |
| hosp senhora da oliveira | hosp senhora da oliveira |
| harvard univ | harvard med sch |
| guys & st thomas nhs fdn trust | guys & st thomas hosp nhs fdn trust |
| univ chicago | univ chicago med |
| hosp st antoine | hop st antoine |
|  | st antoine hosp |
| mayo clin | mayo clin & mayo fdn |
| dartmouth hitchcock med ctr | dartmouth hitchcock ibd ctr |
| cleveland clin | cleveland clin fdn |
| karolinska univ hosp | karolinska univ hosp solna |
| orebro univ | univ orebro |
| amsterdam univ med ctr | amsterdam univ med ctr |
| childrens hosp boston | boston childrens hosp |
| sao joao hosp univ ctr | ctr hosp sao joao |
|  | hosp sao joao |
| guys & st thomas nhs fdn trust | guys & st thomas hosp |
|  | guys & st thomas hosp nhs fdn trust |
| hosp univ mutua terrassa | h mutua terrassa |
|  | hosp univ mutua de terrassa |
| univ leuven hosp | leiden univ med ctr |
| ucl namur | chu dinant godinne ucl namur |
| univ hosp gasthuisberg | univ hosp gasthuisberg |
| univ hosp nancy brabois | univ hosp nancy |
| nancy univ hosp | chru nancy |
| univ hosp schleswig holstein | univ klinikum schleswig Holstein |
| theresienkrankenhaus | theresienkrankenhaus & st hedwig klin |
| ucsf med ctr | ucsf ctr colitis & crohns dis |
| univ Athens | natl & kapodistrian univ athens |
| univ n carolina | univ n carolina chapel hill |
| bnai zion hosp | bnei zion med ctr |
| ctr hosp & univ lille | ctr hosp univ lille |
| erasmus mc | erasmus mc univ med ctr |
| hosp clin san carlos | hosp clin san carlos idissc |
| hosp senhora da oliveira | hosp senhora oliveira |
| hosp univ & politecn la fe | hosp univ & policlin la fe |
| ottawa hosp | ottawa hosp res inst |

**Table S4. Top 44 co-cited publications showed by clusters**

| Document | Cluster |
| --- | --- |
| 1. beaugerie l, 2009, lancet, v374, p1617, doi 10.1016/s0140-6736(09)61302-7 | 1 |
| 1. rutgeerts p, 2005, new engl j med, v353, p2462, doi 10.1056/nejmoa050516 | 1 |
| 1. rahier j, 2014, j crohns colitis, v8, p443, doi 10.1016/j.crohns.2013.12.013 | 1 |
| 1. toruner m, 2008, gastroenterology, v134, p929, doi 10.1053/j.gastro.2008.01.012 | 1 |
| 1. peyrin-biroulet l, 2011, gastroenterology, v141, p1621, doi 10.1053/j.gastro.2011.06.050 | 1 |
| 1. dignass a, 2010, j crohns colitis, v4, p28, doi 10.1016/j.crohns.2009.12.002 | 1 |
| 1. lichtenstein g, 2006, clin gastroenterol h, v4, p621, doi 10.1016/j.cgh.2006.03.002 | 1 |
| 1. long m, 2012, gastroenterology, v143, p390, doi 10.1053/j.gastro.2012.05.004 | 1 |
| 1. rahier j, 2009, j crohns colitis, v3, p47, doi 10.1016/j.crohns.2009.02.010 | 1 |
| 1. gutierrez-dalmau a, 2007, drugs, v67, p1167, doi 10.2165/00003495-200767080-00006 | 1 |
| 1. sandborn w, 2013, new engl j med, v369, p711, doi 10.1056/nejmoa1215739 | 1 |
| 1. feagan b, 2013, new engl j med, v369, p699, doi 10.1056/nejmoa1215734 | 1 |
| 1. gomollon f, 2017, j crohns colitis, v11, p3, doi 10.1093/ecco-jcc/jjw168 | 1 |
| 1. lichtenstein g, 2012, am j gastroenterol, v107, p1409, doi 10.1038/ajg.2012.218 | 1 |
| 1. dayharsh g, 2002, gastroenterology, v122, p72, doi 10.1053/gast.2002.30328 | 2 |
| 1. kandiel a, 2005, gut, v54, p1121, doi 10.1136/gut.2004.049460 | 2 |
| 1. farrell r, 2000, gut, v47, p514, doi 10.1136/gut.47.4.514 | 2 |
| 1. bouhnik y, 1996, lancet, v347, p215, doi 10.1016/s0140-6736(96)90402-x | 2 |
| 1. feagan b, 2000, new engl j med, v342, p1627, doi 10.1056/nejm200006013422202 | 2 |
| 1. connell w, 1994, lancet, v343, p1249, doi 10.1016/s0140-6736(94)92150-4 | 2 |
| 1. lewis j, 2001, gastroenterology, v121, p1080, doi 10.1053/gast.2001.28703 | 2 |
| 1. pearson d, 1995, ann intern med, v123, p132, doi 10.7326/0003-4819-123-2-199507150-00009 | 2 |
| 1. feagan b, 1995, new engl j med, v332, p292, doi 10.1056/nejm199502023320503 | 2 |
| 1. fraser a, 2002, gut, v50, p485, doi 10.1136/gut.50.4.485 | 2 |
| 1. present d, 1980, new engl j med, v302, p981, doi 10.1056/nejm198005013021801 | 2 |
| 1. present d, 1989, ann intern med, v111, p641, doi 10.7326/0003-4819-111-8-641 | 2 |
| 1. candy s, 1995, gut, v37, p674, doi 10.1136/gut.37.5.674 | 2 |
| 1. colombel j, 2004, gastroenterology, v126, p19, doi 10.1053/j.gastro.2003.10.047 | 2 |
| 1. hanauer s, 2002, lancet, v359, p1541, doi 10.1016/s0140-6736(02)08512-4 | 3 |
| 1. colombel j, 2007, gastroenterology, v132, p52, doi 10.1053/j.gastro.2006.11.041 | 3 |
| 1. sands b, 2004, new engl j med, v350, p876, doi 10.1056/nejmoa030815 | 3 |
| 1. targan s, 1997, new engl j med, v337, p1029, doi 10.1056/nejm199710093371502 | 3 |
| 1. hanauer s, 2006, gastroenterology, v130, p323, doi 10.1053/j.gastro.2005.11.030 | 3 |
| 1. podolsky d, 2002, new engl j med, v347, p417, doi 10.1056/nejmra0804647 | 3 |
| 1. present d, 1999, new engl j med, v340, p1398, doi 10.1056/nejm199905063401804 | 3 |
| 1. rutgeerts p, 1999, gastroenterology, v117, p761, doi 10.1016/s0016-5085(99)70332-x | 3 |
| 1. lichtiger s, 1994, new engl j med, v330, p1841, doi 10.1056/nejm199406303302601 | 3 |
| 1. colombel j, 2010, new engl j med, v362, p1383, doi 10.1056/nejmoa0904492 | 4 |
| 1. dhaens g, 2008, lancet, v371, p660, doi 10.1016/s0140-6736(08)60304-9 | 4 |
| 1. baert f, 2003, new engl j med, v348, p601, doi 10.1056/nejmoa020888 | 4 |
| 1. panaccione r, 2014, gastroenterology, v146, p392, doi 10.1053/j.gastro.2013.10.052 | 4 |
| 1. siegel c, 2009, clin gastroenterol h, v7, p874, doi 10.1016/j.cgh.2009.01.004 | 4 |
| 1. vermeire s, 2007, gut, v56, p1226, doi 10.1136/gut.2006.099978 | 4 |
| 1. van ag, 2008, gastroenterology, v134, p1861, doi 10.1053/j.gastro.2008.03.004 | 4 |

**Table S5. Top 43 co-occurrence keywords showed by clusters**

| Keywords | Cluster | Frequency |
| --- | --- | --- |
| management | 1 | 30 |
| diagnosis | 1 | 21 |
| evidence-based consensus | 1 | 21 |
| infection | 1 | 18 |
| opportunistic infection | 1 | 18 |
| risk-factor | 1 | 16 |
| children | 1 | 14 |
| natural-history | 1 | 14 |
| infliximab therapy | 1 | 12 |
| immunomodulator therapy | 2 | 57 |
| rheumatoid-arthriti | 2 | 34 |
| risk | 2 | 31 |
| anti-tnf therapy | 2 | 18 |
| increased risk | 2 | 17 |
| cancer | 2 | 15 |
| population-based cohort | 2 | 13 |
| nonmelanoma skin cancer | 2 | 12 |
| t-cell lymphoma | 2 | 11 |
| infliximab | 3 | 57 |
| maintenance therapy | 3 | 37 |
| efficacy | 3 | 24 |
| infection | 3 | 18 |
| safety | 3 | 18 |
| adalimumab | 3 | 14 |
| combination therapy | 3 | 14 |
| induction | 3 | 10 |
| azathioprine | 4 | 50 |
| 6-mercaptopurine | 4 | 20 |
| maintenance | 4 | 18 |
| anti-tnf therapy | 4 | 18 |
| remission | 4 | 16 |
| methotrexate | 4 | 11 |
| long-term | 4 | 10 |
| randomized controlled-trial | 4 | 10 |
| double-blind | 5 | 36 |
| active crohns-disease | 5 | 14 |
| tumor-necrosis-factor | 5 | 12 |
| placebo-controlled trial | 5 | 11 |
| randomized trial | 5 | 10 |
| surgery | 6 | 12 |
| impact | 6 | 11 |
| postoperative complication | 6 | 10 |
| metaanalysis | 6 | 10 |
